# Profiling alternatively spliced mRNA isoforms for prostate cancer classification

**DOI:** 10.1186/1471-2105-7-202

**Published:** 2006-04-11

**Authors:** Chaolin Zhang, Hai-Ri Li, Jian-Bing Fan, Jessica Wang-Rodriguez, Tracy Downs, Xiang-Dong Fu, Michael Q Zhang

**Affiliations:** 1 Cold Spring Harbor Laboratory, 1 Bungtown Road, Cold Spring Harbor, NY 11724, USA; 2 Department of Biomedical Engineering, State University of New York at Stony Brook, NY 11794, USA; 3 Department of Cellular and Molecular Medicine, University of California, San Diego, La Jolla, CA 92093, USA; 4 Illumina, Inc. San Diego, CA 92121, USA; 5 Department of Pathology, University of California, San Diego, La Jolla, CA 92093, USA; 6 Department of Surgery, University of California, San Diego, La Jolla, CA 92093, USA; 7 VA San Diego Healthcare System, San Diego, CA 92161, USA

## Abstract

**Background:**

Prostate cancer is one of the leading causes of cancer illness and death among men in the United States and world wide. There is an urgent need to discover good biomarkers for early clinical diagnosis and treatment. Previously, we developed an exon-junction microarray-based assay and profiled 1532 mRNA splice isoforms from 364 potential prostate cancer related genes in 38 prostate tissues. Here, we investigate the advantage of using splice isoforms, which couple transcriptional and splicing regulation, for cancer classification.

**Results:**

As many as 464 splice isoforms from more than 200 genes are differentially regulated in tumors at a false discovery rate (FDR) of 0.05. Remarkably, about 30% of genes have isoforms that are called significant but do not exhibit differential expression at the overall mRNA level. A support vector machine (SVM) classifier trained on 128 signature isoforms can correctly predict 92% of the cases, which outperforms the classifier using overall mRNA abundance by about 5%. It is also observed that the classification performance can be improved using multivariate variable selection methods, which take correlation among variables into account.

**Conclusion:**

These results demonstrate that profiling of splice isoforms is able to provide unique and important information which cannot be detected by conventional microarrays.

## Background

Prostate cancer is the second leading cause of cancer illness and death among men in the United States and the third most common cancer world wide [1,2]. According to recent estimates, it accounts for 33% percent of new cancer incidences and six percent of cancer deaths in men world wide [2,3]. In 2002, the number of new incidences and deaths in the United States was approximately 189,000 and 30,200, respectively [2]. The difficulty lies, at least partly, in the heterogeneous nature of the disease. Tumor growth is initially dependent on androgen levels, which stimulate cell proliferation and inhibit apoptosis via the androgen receptor (AR) pathway. The prostate-specific antigen (PSA) level has been a standard screening for early diagnosis; androgen ablation is a prevalent therapy to repress the development of androgen-dependent tumors. However, in many cases, this therapy eventually fails and patients die of the recurrent androgen independent prostate cancer (AIPC), a lethal form that progresses and metastasizes (see reviews in refs [4,5]). Multiple pathways permit cancer cells to escape or bypass the control of the normal AR activation to up-regulate target genes abnormally [6]. Although it has been reported that a number of genes are related to these pathways as well as other aspects of prostate cancer, there is still an urgent need for good biomarkers for early clinical diagnosis and treatment.

Microarray technologies developed in the last decade permit monitoring of mRNA abundance levels of tens of thousands of genes in parallel. The accuracy improvement and cost reduction have made them a routine approach in looking for genes that are differentially expressed between normal and tumor samples or between different tumor types/stages [7-14]. In a recent study, Segal et al. summarized ~2000 array experiments and derived a panoramic view of activated/deactivated gene expression modules for various types of tumors [15].

Microarrays have also been employed in prostate cancer studies. Using cDNA arrays, Dhanasekaran et al. measured gene expression in 50 normal and neoplastic prostate specimens, as well as three prostate-cancer cell lines, and identified gene signatures characterizing androgen-dependent and AIPC samples [16]. Nelson et al. [17] and DePrimo et al. [18] studied gene expression in the androgen treated LNCaP cell line, which was known to be highly androgen responsive. Lapointe et al. profiled 62 primary tumors and 41 normal specimens; three subclasses of tumors representing different tumor stages and risks of recurrence were obtained along with characteristic expression signatures [19]. These studies demonstrated the potential of using microarray analyses in characterizing prostate cancer at the gene expression level.

While transcriptional regulation plays important roles within a cell, post-transcriptional regulation, such as alternative splicing, dramatically increases the diversity of the proteome. Alternative splicing also plays a critical role in gene expression regulation and human diseases [20,21]. It has been reported that about 15% of point mutations that cause human genetic diseases can alter splicing patterns [22]. In particular, splicing aberrations have been characterized in a number of genes and tumor types (see review by Brinkman [23]).

In a previous work, we developed a microarray-based assay called RASL™ (RNA-mediated Annealing, Selection, and Ligation), which can systematically monitor the abundances of unique splicing events [24]. A modified version of the assay, the DASL^® ^(cDNA-mediated Annealing, Selection, extension and Ligation) assay, offers additional robustness for analyzing highly degraded mRNAs, as well as an additional flexibility in probe design [25,26]. Different from other exon-junction arrays [27,28], the DASL assay achieves high specificity and sensitivity due to the fact that both hybridization and ligation of a pair of oligos complementary to the 5' splice site of the upstream exon and the 3' splice site of the downstream exon are required (see ref [25] for details). In our recent study, this technology was applied to profile the abundances of ~1500 unique splice isoforms in prostate cancer cell lines, tumor specimens and normal control samples [29]. This previous study led to two implications: (1) the splicing patterns were altered in a number of genes in response to androgen treatment in the LNCaP cell line; (2) a number of splice isoforms were differentially expressed in tumor samples. They prioritized a list of prostate cancer marker candidates for further investigations. In this study, we extend our previous work and perform a comprehensive analysis of using alternatively spliced isoforms to classify prostate cancer samples. Compared with our previous work, the focus of this study is to quantitatively compare isoform profiling and overall mRNA profiling for cancer classification, which has not been systematically investigated before. To be more specific, the contribution of this study lies in four key aspects: (1) Isoform-sensitive microarrays studies have been assumed to be able to provide more information for cancer classification than conventional microarray studies because isoform abundances couple both transcriptional regulation and splicing regulation. However, it has remained unclear how much unique information could be provided by isoform profiling. In this paper, this assumption is examined qualitatively for the first time through differential expression analysis. Further examinations for several genes are also described. (2) As in a number of other microarray studies (e.g. [16,19]), hierarchical clustering has been used to segregate similar tissues. This approach was not able to obtain an unbiased estimation of the predictive power for new unknown samples. To assess the predictive power of isoform profiling and that of overall mRNA profiling, a support vector machine with recursive feature elimination (SVM-RFE) was employed to build prediction models and the prediction accuracies were compared. (3) Building a prediction model with a minimal subset of variables is one of the critical tasks in cancer classification. We compared two different variable selection methods for sample classification and examined whether the robustness of prediction can be improved by taking the correlation among isoforms into account during variable selection. (4) In our previous study, two smaller datasets generated in different batches were analyzed separately. The two lists of candidate markers selected from the two datasets had a relatively small overlap. To achieve more robust results, all analyses in this study were based on the larger combined dataset after careful normalizations.

## Results

In our previous work [29], the two datasets of prostate tumors and normal samples were analyzed separately by hierarchical clustering because they were generated in two different batches and there were significant heterogeneities between them (data not shown). In both datasets, splice isoforms could be used to separate tumor samples and normal samples. However, the sample size in each dataset was limited and the overlap between the two lists of differentially expressed isoforms selected from the two datasets was relatively small. In this paper, the two datasets were combined after careful normalizations to achieve more robust results and statistical power (see Methods). The combined datasets included 22 cases of prostate tumors and 16 matched normal samples.

### Splice isoforms reveal distinct signatures of prostate cancer

**Figure 1:**
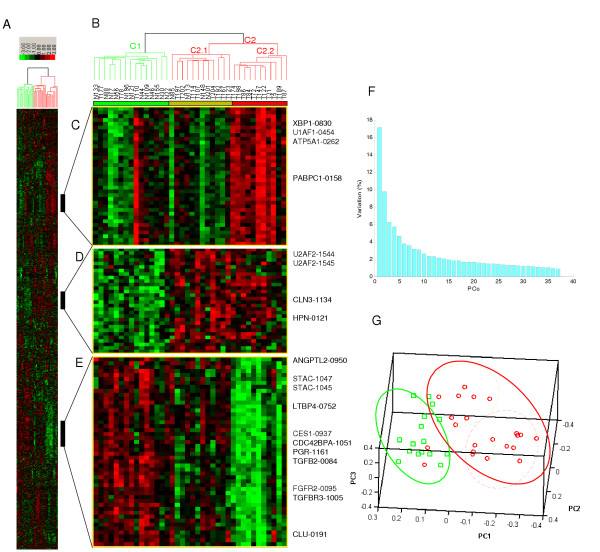
**Prostate tumor and normal samples can be separated into distinct groups. (A) **A thumbnail overview of the result of the two-way average-linkage hierarchical clustering of 38 arrays (columns) and 1532 isoforms (rows), as described in ref [30]. (**B) **Zoom-in view of the array clustering dendrogram. The two array clusters, C1 and C2, are enriched by normal samples and tumor samples, respectively. Cluster C2 is formed by two sub-clusters, reflecting differences in tumor percentage and stroma. (**C-E) **Isoform signatures up- or down-regulated in different array clusters. (**F and G) **The result of SVD. (**F**) The percentage of variation (y-axis) captured by each principal component (x-axis). (**G) **The low dimensional projection of arrays in the 3D space spanned by the first three principal components. SVD identified the same hierarchical structure as revealed by hierarchical clustering.

We first examined whether the global distinction between tumors and normal samples still exists in the combined dataset by unsupervised methods. As expected, tumors can be readily separated from normal samples by average-linkage hierarchical clustering (Figure 1A and 1B, cluster C1 and C2) [30,31]. Compared with cluster C2, the majority of tissues in cluster C1 are normal prostate and stroma, with the average tumor percentage being 8.2% (p < 0.0001), and stromal percentage being 63.4% (p < 0.0001). Of the three tumors segregated with normal samples in cluster C1, two have low tumor content. Additional analysis reveals that C2 cases in general have a significantly higher percentage of more advanced stages (Stage 3 or above) and more patients die of prostate cancer compared to C1 cases. Specifically, 100% of the cases in C1 were from patients with organ confined tumors (stage T2), whereas 50% of the cases in C2 were from metastasized patients (stage T3 tumors, p < 0.001). At the time of analysis, none of the C1 patients died of prostate cancer while14% of the C2 patients died of prostate cancer. Interestingly, the cluster C2 enriched by tumors was further segregated into two sub-clusters, reflecting different percentage in tumor and stromal content (Mean tumor content in sub-cluster C2.1 = 47.9% v.s. C2.2 = 64.5%, p = 0.1; Mean stromal content in C2.1 = 35.8% v.s. C2.2 = 20.5, p = 0.04).

Singular value decomposition (SVD) was used to identify an orthogonal low dimensional space which preserves the maximal variation of the original high dimensional space. The first two principal components capture 17% and 9% of the total variation, respectively (Figure 1F). Remarkably, the first principal component alone shows a strong separation of tumor and normal samples. The clusters and sub-clusters derived from hierarchical clustering are also reflected in the 3D space spanned by the first three principal components (Figure 1G), which confirms the results of clustering.

Further examination of the gene clustering results shows distinct molecular signatures of different tissue clusters, including both well known marker genes and less studied marker candidates (Figure 1C, D and 1E). Figure 1C shows isoforms up-regulated in cluster tumor sub-cluster C2.2, including isoforms from genes RPS2, XBP1, U1AF1 and ATP5A1, all of which were known to be up-regulated in tumors. Figure 1D shows isoforms down-regulated in normal tissues and up-regulated in tumor tissues, including isoforms from genes U2AF2, CLN3 and HPN. Figure 1E shows isoforms with high expression levels in normal tissues and down-regulated in tumor tissues, especially in sub-cluster C2.2. Several genes in this cluster are known to be involved in the TGF-beta signaling pathway, such as TGFB2, LTBP4 and TGFBR3.

### Differentially expressed splice isoforms

A two sided t-test was used to identify genes with statistically significant changes in expression between tumors and normal samples. A false discovery rate (FDR) or q-value was calculated as described previously [32], to correct for multiple testing. As a result, 464 isoforms (30%) representing 222 genes (61%) are reported as being significant (q-value < 0.05) [see Additional file 1]. The high proportion of differentially expressed isoforms reflects the fact that the genes profiled are potentially related to prostate cancer according to existing evidence. Top isoforms among them include AMACR-2094, FGFR2-0101, FGFR2-0097, FGFR2-0098, CLU-0192, PGR-1162, etc.

### Profiling of splice isoforms provides additional information to overall mRNA abundances

In theory, profiling individual splice isoforms can provide more information than profiling overall mRNA levels as in conventional microarrays. This is because isoform profiling detects the combinatorial effects of both transcriptional regulation and splicing regulation. Consider the simplest case of a gene with two alternatively spliced isoforms. If one isoform is up-regulated in tumors whereas the other is down-regulated, the overall mRNA abundance may not change. On the contrary, if the overall mRNA level is differentially expressed, there is at least one isoform exhibiting differential expression. However, how much additional information can be obtained for cancer classification by isoform profiling has not been systematically evaluated. To address this question, we compared individual isoforms and overall mRNAs for differential expression.

**Figure 2:**
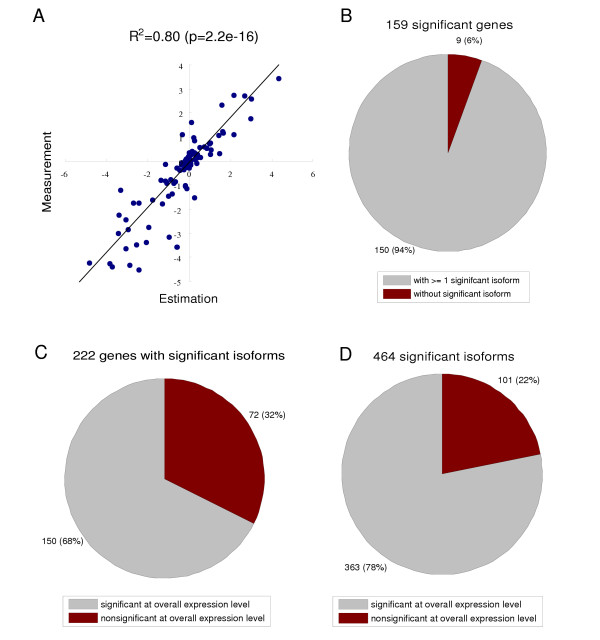
**Profiling splice isoforms provides additional useful information for prostate cancer classification. (A) **The validity of estimating the overall mRNA abundance level from the isoform abundance level. The overall mRNA level was estimated by summing up the abundances of individual isoforms for each gene. The estimated mRNA abundances of 107 genes were compared with direct measurements by an independent expression microarray design (described in main text). Plotted are the scatter-plot of log expression ratios of these genes in two prostate cancer cell lines, LNCaP and PC-3. These two approaches show good agreement (*R*^*2 *^= 0.80, p = 2.2e-16). (**B) **159 genes out of 364 profiled genes in the DASL assay exhibit differential expression between tumors and normal samples at the overall mRNA level (q-value = 0.05). Most of them (92%) have isoforms with significant differential expression. (**C and D) **464 isoforms from 222 genes are reported as being differentially expressed between tumors and normal tissues (q-value = 0.05), which may be prostate cancer marker candidates. 32% of these genes (corresponding to 22% significant isoforms) do not show differential expression at the overall mRNA level, therefore can not be detected by conventional microarrays.

Due to the costs and array capacity, the original array design did not include probes targeting common regions of all isoforms. Therefore, the overall mRNA expression level can not be obtained directly. However, since the probed exon junctions target unique major isoforms and hybridization efficiencies of different probes are comparable [25], we reason that the overall expression level can be estimated by summing up the abundances of individual isoforms. To examine the validity of this idea, two well-known prostate cancer cell lines LnCaP and PC-3 were profiled using the same DASL assay (splicing array). For comparison, 107 genes were arbitrarily selected for gene expression profiling in the same cell lines (expression array). An independent oligo pool targeting common regions of all isoforms in each of the 107 genes were used in the expression array. Therefore, the log expression ratio of each gene in the two cell lines can be obtained from the estimation based on the splicing array and from the direct measurement in the expression array independently. To our satisfaction, the two quantities are highly correlated (*R^2 ^*= 0.80, , p = 2.2e-16), suggesting a reasonable accuracy of the estimation (Figure 2A).

Having validated the approach, the overall mRNA abundances of each gene in prostate tissues were estimated. A t-test was similarly applied to identify genes with significant differential expression in tumors at the overall mRNA level. In total, 159 genes (43.6%) are reported as being significant (q-value < 0.05). Again, the high proportion of significant genes reflects the fact that they are potentially relevant to prostate cancer according to previous studies. Strikingly, more genes are called significant by examining individual isoforms than by examining overall mRNAs (222 vs 159, p = 0.001, chi-square test). Among the 159 genes that are called significant, 150 genes (94%) have at least one isoform that is reported as significant (Figure 2B). In contrast, only 68% of genes with significant isoforms can be detected at the overall mRNA level. The remaining 32% of the genes have significant isoforms but do not exhibit significant differential expression at the overall mRNA level. It is important to note that these genes represent the unique information that is provided by splice isoform sensitive microarrays and cannot be obtained from conventional microarrays.

From the perspective of isoforms, 78% of significant isoforms are from those genes that are also called significant whereas 22% of significant isoforms are from those genes that do not show overall mRNA differential expression (Figure 2D) [see Additional file 2 and 3]. Multiple testing has been appropriately accounted for, so the additional significant calls using splice isoforms are not due to the different stringencies of thresholds, but reflect additional information provided by including splicing regulation.

For many genes, only one isoform is specifically altered in tumors. In these cases, the addition of other isoforms to the total mRNA level simply introduces random noise. Notably, there are 14 genes with one isoform being up-regulated in tumors and another isoform being down-regulated. Among them, 3 genes are not significant at the overall mRNA level: CD44 (CD44-1404 vs CD44-1570), ITGB1 (ITGB1-0032 vs ITGB1-0033) and MAPT (MAPT-1060 vs MAPT-1061). CD44 is a multifunctional receptor involved in cell-cell interactions and cell trafficking. Deregulated expression of a number of variants is correlated with tumor metastasis (reviewed by [23]). ITGB1 is a protein involved in extra-cellular matrix interactions and is also related to many tumor types, including prostate cancer [22].

There are relatively fewer studies discussing the role of MAPT in cancer. MAPT encodes the microtubule-associated protein tau mainly expressed in the central nervous system. Mutations in the MAPT gene disrupt the normal binding of tau to tubulin. This in turn results in pathological deposits of hyperphosphorylated tau in the brain, which is a pathological hallmark of several neurodegenerative disorders (see review by Rademakers et al. [33]). Previously, Sangrajrang et al. found that MAPT was also expressed in the DU145 cell line using RT-PCR and the expression at the protein level was validated by Western blotting [34]. The expression was elevated after estramustine treatment and the authors suggested that the protein may be positively related to drug resistance. This was consistent with a recent report demonstrating that the up-regulation of the protein tau was correlated to the decrease of paclitaxel sensitivity in breast cancer [35]. In our data, MAPT-1060 (representing the skipping of exon 4A, numbered according to ref [33]) has a two fold increase in tumors relative to normal tissues(q-value = 0.86%), whereas MAPT-1061 (representing the inclusion of exon 4A) has a two fold decrease in tumors relative to normal tissues (q-value = 0.16%). It is likely that exon 4A is uniquely skipped in prostate cancer cells. This hypothesis is further supported by the following evidence. Exon 4A harbors a C/T single nucleotide polymorphism (SNP) near the 5' splice site (Entrez SNP: rs17651549, contig position: 2715394). This SNP was assayed from 71 individuals and the C/T ratio is 0.886/0.114. In the major C allele, a putative exonic splicing enhancer (ESE) *cagccgg *encompassing the SNP is predicted by ESEfinder and resembles the specific RNA binding site of SF2/ASF, a critical serine rich (SR) protein that helps to recruit the splicing apparatus (score: 4.6, threshold: 1.956) [36]. This putative ESE is disrupted in the minor T allele for all four SR proteins in ESEfinder including SF2/ASF, SC35, SRp40 and SRp55. However, further experimental studies and confirmation of the splicing alteration may be required to validate this hypothesis.

### Profiling of splice isoforms improves predictive power

A robust prediction model to classify unknown samples is essential for early cancer detection and diagnosis. Having demonstrated that a large fraction of genes show differential expression at the splice isoform level but not at the overall mRNA level, a key question is how much additional predictive power can be achieved by isoform profiling. Another related problem is to select minimal subsets of variables with the best performance. Like many other types of tumors, a single molecular marker is usually not robust enough for prostate cancer detection, as is the case for the widely used PSA level for early stage screening. At the other extreme, including all variables from a genome-wide profiling is not justifiable either, due to the noise introduced by a huge number of uninformative variables and the difficulty in the interpretation of the resulting model.

A support vector machine (SVM) was used here to build the classifier because of its excellent performance in many previous studies with small sample sizes [37]. An recursive feature elimination (RFE) algorithm was integrated as described previously with minor adaptations [38].

**Figure 3:**
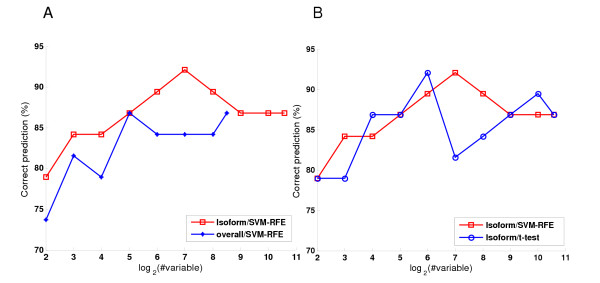
The performance is measured by leave-one-out cross validation. To get unbiased result, the variable selection and training are done in training arrays, which is completely independent with the testing array. (**A) **The comparison in classification performance of SVM-RFE selected variables using individual isoforms and the overall mRNAs. (**B) **The comparison in classification performance of variable subsets selected by SVM-RFE and t-test, using individual isoforms.

Leave-one-out cross validation (LOOCV) with external variable selection was used to give an unbiased evaluation of the prediction accuracy (see Methods for details). SVM-classifiers were built using the individual splice isoforms and estimated overall mRNA abundances. The results of LOOCV are shown in Figure 3A. For the classifiers using isoform abundances, the best performance, 35 correct predictions out of 38 samples (92%), is achieved when 128 isoforms are included for classification. For the classifiers using overall mRNA abundances, the best performance (87% correct predictions) is achieved when 32 genes are used. The additional information provided by splicing regulation gives rise to an improvement of about 5% in predictive power. Importantly, the difference persists in the whole range of different sizes of selected variable subsets, which is unlikely by random chance. With an independent method, this demonstrates that isoform profiling can provide valuable information for cancer classification. Also, the classification performance deteriorates when the subset of selected variables is too small in size (e.g., 4 variables). This is consistent with the previous observation that a robust cancer prediction model should use a reasonable number of molecular signatures [39].

### Comparison of different variable selection methods

Both t-tests and SVM-RFE can generate lists of candidate markers. These two approaches represent univariate variable selection and multivariate variable selection, respectively. They have different assumptions and may characterize different yet overlapping perspectives of the molecular mechanisms underlying the data. For example, variables are assumed to be independent in a t-test but there is no assumption of independence in SVM-RFE. Comparing the multiple outputs of selected signatures by different methods may shed further insights into the data and the methods. Therefore, the two different variable selection approaches, t-test and SVM-RFE, were applied to select marker candidates and their performances in building linear SVM models were compared. The results of LOOCV are shown in Figure 3B. The best performance of t-test selection is achieved with a similar number of variables as SVM-RFE. Both methods result in an accuracy of 92%. The similar best performance by t-test and SVM-RFE is likely due to the distinct features of tumors and normal tissues. The information to classify the two groups is largely redundant. However, the curve of prediction accuracy by the SVM-RFE selection is smoother than that by the t-test selection as the size of selected variable subset decreases. This smaller variation suggests that SVM-RFE is more robust than t-test in variable selection for cancer classification.

The 128 isoforms selected by t-test (t-test128 list) and the 128 isoforms selected by SVM-RFE (svm128 list) share 42 isoforms (Table 2). The common list includes AMACR-2094, AMACR-2097, AMACR-2098, FGFR2-0099, FGFR2-0094, PGR-1166 and PGR-1555 among others. They may represent robust marker candidates. Significant isoforms in each list were further divided into two groups according to whether the corresponding genes also exhibit significant differential expression at the overall mRNA level. Interestingly, among those 86 isoforms included only in the svm128 list, 13 of the isoforms are in the category that the corresponding genes do not show significant differential expression at the overall mRNA level. In contrast, among the 86 isoforms included only in the t-test128 list, only 4 isoforms lie in this category. Therefore, SVM-RFE captures more information uniquely provided by considering splicing regulation (p = 0.03, chi-square test). This demonstrates the advantage of a variable selection method taking the correlation between variables into account.

## Discussion

The diagnosis and treatment of prostate cancer are fields with long histories. Various efforts have led to the progressive understanding of the disease. However, the present criteria of diagnosis and prognosis, as well as the approaches of treatment and surgery, are not sufficiently reliable. Previous gene expression profiling studies on prostate tumors and normal tissues demonstrated the feasibility in characterizing the molecular alterations at the overall mRNA transcript level. However, these transcriptome analyses were based on the old central dogma of "one gene, one mRNA", which may underestimate the complexity of tumorigenesis [23].

Previously, we carried out a study of prostate cancer by exon-junction microarray-based assay and demonstrated the power of this integrated technology in detecting both transcriptional and splicing regulation [25,29]. In this paper, we present systematic analyses with the focus on using splice isoform profiling for prostate cancer classification. Isoform-sensitive microarrays have been used in several recent studies [24,25,27,29,40-44] (also see review by Lee and Roy [45]). These studies demonstrated that isoform-sensitive microarray is a reliable, high throughput approach to detecting splicing alterations in various tissues and conditions. Although more and more data are expected to be generated in the near future, the dataset used in this study is the only dataset currently available which screened a relatively large sample of cancer and normal tissues. As far as we know, this is the first systematic comparison of isoform-sensitive microarrays and conventional microarrays for cancer classification.

Previous studies have used a "splice index", which is the fraction of each isoform, to remove the effect of transcriptional regulation [40,41]. This is not desired for cancer classification because as much information as possible should be incorporated. Therefore the abundance of each isoform, which couples both transcriptional regulation and splicing regulation, was used for classification. The performance was compared with that of using overall mRNA abundances. One has to note a caveat of the current DASL assay: it does not include probes complementary to the common regions of all mRNA transcripts for each gene due to the current limit in array capacity. Therefore, the overall mRNA level was estimated indirectly by summing up all the isoforms targeted. The estimation is not ideal due to the fact that not all isoforms were included in the array and the probes target splicing events that are not mutually exclusive in several cases. However, the estimation is reasonably good and highly correlated with the direct measurement by an expression array. Various other methods were tried to estimate the overall mRNA abundances, but the method used here is the most accurate and simplest.

Among the ~1500 isoforms from putative prostate cancer-related genes, a large fraction of them exhibit differential expression in cancer cells. Tumors and normal tissues can be readily separated by both unsupervised and supervised methods. By comparing individual isoforms and overall mRNAs for differential expression, we arrived at the conclusion that an isoform-sensitive microarray, which detects coupled transcription and splicing regulation, can provide about 30% more information than conventional microarrays. This value may still be underestimated due to the following reasons. The current DASL assay included only 364 genes potentially relevant with prostate cancer derived from previous studies. Till now, a large body of literature, especially those in the genomic scale, focused more on transcriptional regulation. Therefore, the selection of genes may be biased to those exhibiting aberrant transcriptional regulation.

The optimal prediction model was built by SVM with variable selection integrated, a powerful machine learning approach. With around 100 isoforms, the best classification performance can be achieved at a correct prediction rate of 92%. Compared with the optimal SVM classifier built with overall mRNA abundances, this represents an improvement of five percent. Therefore, both differential expression analysis and classification analysis quantitatively demonstrated the advantage of isoform-sensitive microarrays.

We also compared the effect of different variable selection approaches on classification performance. By taking the correlation between isoforms into account, isoforms selected by SVM-RFE are more robust for classification than isoforms selected by a t-test. Although univariate two-sample comparisons such as t-test are widely used to identify differentially expressed genes, the assumption of independence between genes or isoforms is not biologically justifiable. In cancer signal transduction pathways, a group of genes in the same pathway are interacting with each other; cross-talks often exist between pathways as well (C Jiang, personal communication). Variables are more convoluted in the DASL data due to the coupling of transcription and splicing. The multi-loci nature of the disease also makes it difficult to use a single or few molecular markers to build a sufficiently robust prediction model.

This study identified a number of known prostate cancer markers as well as less studied marker candidates, which span a wide spectrum of biological functional roles. Some are related to signal transduction (SIM2 and CDC42BPA), as well as extracellular matrix and cytoskeleton (CD44, MAPT and ILK). Others appear to be involved in epidermal differentiation and proliferation (KRT15, IGF1, PGR and HPN), cell growth and development (FGFR2), apoptosis (DBCCR1 and CLU), lipid metabolism (AMACR), etc. Very significantly, multiple isoforms from AMACR, a key player in catalyzing the isomerization of alpha-methyl-branched fatty acid and a recently reported good prostate cancer marker, show the strongest signal in our data [46]. Several genes encoding splicing factors, such as U2AF1, U2AF2 and DHX34, also show significant differential expression. This is consistent with our observation that a large fraction of splicing factors are deregulated in tumors (C. Zhang et al, unpublished data).

Another interesting observation obtained by examining the panel of potential marker candidates selected by one or more methods is that a number of genes are normally expressed specifically in neuronal cells (such as MAPT, STAC, NELL2, etc). The relationship between abnormal expression of neuronal genes and tumors is not completely clear. However, it is believed that there is a link between diverse neurodegenerative diseases and cancers via the induction of antitumor immunity, known as paraneoplastic neurological degenerations (PND) (see review by Albert and Darnell [47]). Alternative splicing is also prevalent for neuronal genes.

## Conclusion

Profiling of individual isoforms can provide unique and important additional insights into prostate cancer classification. Robust prediction models can be built with a subset of isoforms selected by multivariate variable selection method.

## Methods

### DASL assay

The DASL assay and array hybridization were described previously [25]. In contrast to conventional microarrays which only measure the overall mRNA abundance of each gene, the most distinguishing feature of the DASL assay is that it permits the profiling of each individual mRNA splice isoform quantitatively. This technology has been shown to be highly sensitive, specific and reproducible (*R*^*2 *^> 0.99 between replicates).

### Tumor and normal tissue profiling

The array used in this study included 1532 isoforms from 364 genes. These genes, potentially related to prostate cancer, were selected from published literature, previous microarray data analysis, human genome anatomy projects and EST searching. All of them have known gene structures and alternative splicing patterns. Alternatively spliced exon junctions probed in the array were obtained by the alignment of mRNA transcripts/ESTs and the genome. They were manually annotated and are publicly available from the MAASE database [48,49]. In total, 22 cases of archived formalin fixed, paraffin embedded prostate tumors at different tumor stages and 16 adjacent normal matching samples from the UCSD prostate tumor bank were assayed, each with two replicates (Table 1). The detailed information about sample collection, preparation, RNA profiling experiment and probe quantification were described elsewhere [29]. The raw data is available from the authors upon request.

### Microarray data normalization and statistical analysis

Before further analysis, a log_2 _transformation was applied to raw intensities. Since the dataset was generated in two batches, heterogeneity between batches has to be removed. As a first step, each isoform (row) inside each batch was median-centered separately. Then, the two batches were combined and standardized to unit variance across each array (column) and isoform (row) as a whole. Finally, the two replicates of each tissue sample were averaged. In this way, each value in the data matrix represents the log expression ratio of an isoform in a particular sample with respect to a "common control" [15]. The effect of normalization was examined by clustering the combined data using real expression values and null control probes, respectively. After normalization, there is no visible artificial distinction between the two batches.

To estimate the overall mRNA abundance of each gene, the intensities of all isoforms were summed. Then the same log transformation and normalization steps above were applied. Again, each normalized value represents the log expression ratio of mRNA abundance in a particular sample with respect to a "common control".

A two-sided t-test was used to select isoforms or genes with significant differential expression between tumors and normal tissues. To correct for the effect of multiple testing, false discovery rate (FDR) or q-value was calculated as described previously [32].

A chi-square test was used to analyze the significance of frequency data.

### Singular value decomposition

Singular value decomposition (SVD) is a standard mathematical transformation to find a set of orthogonal principal components (PCs) which explain as much variation as possible [50]. The power of SVD has been shown in many fields as well as in microarray data analysis. Alter et al. and Holter et al. suggested that the first two PCs can characterize cell cycle phases of yeast genes[51,52]. Liu et al. separated prostate and colon tumors from others with the first PC alone[53]. In a similar spirit, SVD transformation was used in this study to reveal the "hidden" information underlying the original high dimensional dataset.

### SVM-RFE

A linear support vector machine (SVM) optimizes a linear classifier *D *(**x**_*i*_) = **w·x_*i *_**+ *b *by maximizing the margin of support vectors from two classes, where **x**_*i *_is the expression vector of a sample ***i ***and **w **is the vector of weighting coefficient, reflecting the contribution of each variable in classification [37]. In the past few years, SVM has been developed and shown as a powerful tool for classification problems with a small sample size, such as microarray sample classification (e.g. ref [7]). SVM-RFE (RFE stands for recursive feature elimination) is a wrapper approach of variable selection, in which the predictive power of a subset of variables is measured collectively by the accuracy of the classification based on the subset in consideration [38,54]. Since an exhaustive search of the optimal subset is a combinatorial problem, a heuristic strategy must be applied. In SVM-RFE, variables are ranked by the weighting vector **w**, by which a subset of variables with top ranks is selected. Then the weighting vector **w **is re-evaluated by optimizing a new classifier with the selected subset and a smaller subset is selected therein. This recursive procedure continues until the subset is small enough or the classification performance approaches some criteria. In this way, informative variables for classification are recursively selected (or uninformative variables are recursively eliminated). Details of the algorithm can be found in ref [38]. Our implementation of SVM-RFE used SVMTorch for linear SVM model calculations [55]. The default soft margin (C = 100) was used.

### Cross validation incorporating variable selection

Due to the limited sample size, leave-one-out cross validation (LOOCV) was used to evaluate the classification performance of SVM classifiers built with subsets of variables selected by t-test and SVM-RFE. In each round, one array (test set) is left out to test the classifier trained on the remaining arrays (training set). The classification performance is the percentage of correct predictions in all rounds. To get an unbiased result, in each round the variable selection step must be applied "externally", i.e. only on the training set, excluding the sample left out for validation [39]. Therefore, the subsets of variables selected might be different from round to round. The number of times that a variable is selected reflects the robustness of the variable for classification. Therefore the final subset of variables can be selected by ordering the number of times that a variable is included in the selected subsets of all rounds.

## Authors' contributions

CZ and HRL carried out data analysis. JBF designed the microarray. JWR collected clinical samples. TD built the database of pathological information. XDF, JBF and MQZ participated in the design of this study. CZ and MQZ drafted the manuscript.

## Supplementary Material

Additional File 1 Supplementary table S1. Splice isoforms differentially expressed between prostate cancer and normal samples (q-value<0.05). Click here for file

Additional File 2 Supplementary table S2. Significant isoforms from those genes that are also called significant at overall mRNA level (q-value<0.05). Click here for file

Additional File 3 Supplementary table S3. Significant isoforms from those genes that are not significant at overall mRNA level (q-value<0.05). Click here for file

## Figures and Tables

**Table 1:** Pathological information of tumor and normal prostate samples

**ID**	**Age**	**Risk group**	**% tumor**	**BPH**	**Atrophy**	**Stroma**	**Inflam**	**PSA**	**Gleason**	**Stage**
T5	67	low	50	0	0	20	0	8.48	5 + 4 = 9	T3bN1Mx
T21	74	Low	60	10	10	20	0	6.7	4+4 = 8	T2bNxMx
N22	74	Low	0	10	40	50	0	6.7		T2bNxMx
N30	55	Int	0	10	30	68	0	11.68		T2bN1Mx
N44	61	low	0	10	2	88	0	5.46		T2cNxMx
N46	74	High	0	45	20	35	0	8.06		T2aNxMx
N56	67	High	0	5	0	94	0	5.7		T2aN0Mx
T72	68	Int	70	0	0	30	0	8.27	4+3 = 7	T3bN1Mx
N77	66	Int	0	0	10	89	1	3.15		T2cNxMx
T78	66	Int	35	5	5	55	0	3.15	3+4 = 7	T2cNxMx
T84	60	high	70	5	0	25	0	9.99	4+5 = 9	T3bN0Mx
N85	66	Int	0	30	0	70	0	4.37		T3bN0Mx
T86	66	Int	90	5	0	5	0	4.37	4+4 = 8	T3bN0Mx
T87	61	High	25	45	5	25	0	2.23	4+3 = 7	T2bN0Mx
N88	61	High	0	10	30	60	0	2.23		T2bN0Mx
T107	68	Int	60	10	0	30	0	7.4	4+3 = 7	T2bNxMx
N109	67	Low	0	5	0	90	5	7		T2bNxMx
T110	67	Low	40	0	0	58	0	7	3+4 = 7	T2bNxMx
N113	70	Low	0	10	5	85	0	4.78		T3aNxMx
T114	70	Low	40	0	5	55	0	4.78	4+4 = 8	T3aNxMx
N121	50		0	30	2	68	0	0.22		
T122	67	Low	70	0	5	25	0	7	3+4 = 7	T2bNxMx
T123	78		80	0	0	20	0	17.7	5+5 = 10	NR
N133			0	25	5	75	0			
T147	78	Int	70	0	0	30	0	6.9	4+4 = 8	T2bNoMx
N148	67	Low	0	35	10	55	0	4.68		T2aNxMx
N155	70	Int	0	40	10	48	2	8.4		T2cNxMx
T167	72	Int	80	0	10	10	0	18	4+4 = 8	T2bNoMx
T174	83	high	70	5	0	25	0	15	5+4 = 9	T4
T177	67	Int	40	0	30	30	0	10.87	4+4 = 8	T2cNoMx
T189	77	N/A	70	0	0	0	30	2.51	5+5 = 10	T2bN2Mx
T192	61	Int	50	5	10	35	0	5.7	4+4 = 8	T3aNxMx
N196	73	low	0	40	5	55	0	4.59		T2bNxMx
T197	67	high	95	0	0	5	0	21.82	4+4 = 8	T3aN1Mx
T198	60		60	0	10	25	0	4.06	4+4 = 8	T3bNxMx
N201	64		0	20	5	45	0	UNK		T2bNxMx
T202	67	Int	90	0	5	5	0	12.34	4+4 = 8	T3bNxMx
T204	54	low	80	0	5	15	0	3.91	4+5 = 9	T3cNxMx

**Table 2:** Top prostate cancer marker candidates selected by both t-test and SVM-RFE.

**Isoform ID**¶	**Normalized log2 expr**	**FDR# (q-value)**	SVM-RFE freq.*	**Protein Name**§
ALDH1A2-0004	-1.21	1.3E-04	35	Aldehyde dehydrogenase 1A2
AMACR-2094	1.41	6.7E-05	38	Alpha-methylacyl-CoA racemase
AMACR-2097	1.08	9.2E-04	38	
AMACR-2098	0.99	1.8E-03	17	
ANXA2-0914	-1.04	1.8E-03	36	Annexin A2
APBB3-0185	1.01	1.5E-03	38	Amyloid beta (A4) precursor protein-binding family B member 3
BC008967-0877	-1.38	7.9E-05	26	
C21ORF5-0239	1.24	6.0E-04	35	Chromosome 21 open reading frame 5
C7ORF24-0062	1.30	8.4E-05	17	
CALCR-1180	1.05	5.2E-04	37	Calcitonin receptor
CCT8-0334	1.21	1.5E-04	32	Protein with high similarity to C. elegans Y55F3AR.3
CDC42BPA-1048	-1.19	6.0E-04	38	CDC42 binding protein kinase alpha
CDK7-0899	1.35	8.4E-05	37	Cyclin-dependent protein kinase 7
CES1-0937	-1.34	7.9E-05	32	Cat eye syndrome chromosome region candidate 1
CLU-0197	-1.11	1.2E-03	38	Clusterin (apolipoprotein J)
EDNRB-1187	-1.24	4.7E-04	26	Endothelin type B receptor
FGFR2-0094	-1.13	4.0E-04	19	Fibroblast growth factor receptor 2
FGFR2-0099	-1.03	7.7E-04	28	
HEBP2-0472	1.08	7.8E-04	24	Heme binding protein 2 (placental protein 23)
HSPD1-0152	1.10	1.8E-03	37	Chaperonin 60
HSPD1-0154	1.17	2.8E-04	31	
IGSF4-0722	0.72	2.1E-03	38	Immunoglobulin superfamily member 4
IMPDH2-0144	1.25	1.3E-04	34	Inosine monophosphate dehydrogenase type 2
IQGAP2-0234	1.17	5.6E-04	22	IQ motif containing GTPase activating protein 2
LAMR1-0523	1.20	1.3E-04	38	Laminin receptor 1
LTBP4-0746	-1.27	1.5E-04	33	Latent transforming growth factor beta binding protein 4
LTBP4-0748	-1.10	1.4E-03	38	
LYPLA1-0860	1.38	7.9E-05	35	Lysophospholipase 1
NELL2-0805	-1.10	1.2E-03	24	Nel-like 2
PGR-1166	-1.16	4.0E-04	32	Progesterone receptor
PGR-1555	0.85	7.5E-04	38	
PPIB-0969	0.94	2.2E-03	34	Cyclophilin B
PTS-0059	-1.07	2.2E-03	31	6-pyruvoyltetrahydropterin synthase
PYCR1-0058	1.28	4.1E-04	38	Pyrroline-5-carboxylate reductase 1
RING1-0217	-0.93	1.7E-03	22	Ring finger protein 1
SFRS10-1126	0.95	2.0E-03	34	Splicing factor arginine/serine rich 10
SMPDL3B-2030	1.09	2.2E-04	38	Protein containing a calcineurin-like phosphoesterase domain
STAC-1044	-1.31	7.9E-05	34	Src homology three and cysteine rich domain
TGFB2-0085	-1.11	6.5E-04	38	Transforming growth factor beta 2
TRIM29-1350	-1.29	1.5E-04	35	Ataxia telangiectasia mutated
TRIM29-1353	-1.20	1.7E-04	34	
TXNIP-1116	1.09	1.3E-03	38	Thioredoxin interacting protein

¶ detail information of each isoform, such as the exon junction and probe design, can be accessed at the MAASE database [48];

# FDR is calculated using all 38 samples;

§SVM-RFE freq.: the number of times that an isoform is included in 38 selected subsets in leave-one-out cross validation.
